# On the Regression Model for Generalized Normal Distributions

**DOI:** 10.3390/e23020173

**Published:** 2021-01-30

**Authors:** Ayman Alzaatreh, Mohammad Aljarrah, Ayanna Almagambetova, Nazgul Zakiyeva

**Affiliations:** 1 Department of Mathematics and Statistics, American University of Sharjah, Sharjah P.O. Box 26666, United Arab Emirates; 2 Department of Mathematics, Tafila Technical University, Tafila 66110, Jordan; aljarrah@ttu.edu.jo; 3 Department of Mathematics, University of Amsterdam, 1098 XH Amsterdam, The Netherlands; ayanna.almagambetova@nu.edu.com; 4 Zuse Institute Berlin, 14195 Berlin, Germany; zakiyeva@zib.de

**Keywords:** *T*-*X* family, logistic distribution, normal distribution, moments, estimation, regression

## Abstract

The traditional linear regression model that assumes normal residuals is applied extensively in engineering and science. However, the normality assumption of the model residuals is often ineffective. This drawback can be overcome by using a generalized normal regression model that assumes a non-normal response. In this paper, we propose regression models based on generalizations of the normal distribution. The proposed regression models can be used effectively in modeling data with a highly skewed response. Furthermore, we study in some details the structural properties of the proposed generalizations of the normal distribution. The maximum likelihood method is used for estimating the parameters of the proposed method. The performance of the maximum likelihood estimators in estimating the distributional parameters is assessed through a small simulation study. Applications to two real datasets are given to illustrate the flexibility and the usefulness of the proposed distributions and their regression models.

## 1. Introduction

Existing distributions do not always provide an adequate fit. Hence, generalizing distributions and studying their flexibility are of interest for researchers over recent decades. One of the earliest works on generating distributions was done by [1] who proposed a method of differential equation as a fundamental approach to generate statistical distributions. Ref. [2] also made a contribution in this category and developed another method based on differential equation. After that, other methods were developed such as the method of transformation [3] and the method of quantile function [4,5]. More recent techniques in generalizing statistical distributions emerged after the 1980s and can be summarized into five major categories [6]; the method of generating skew distributions, the method of adding parameters, the beta generated method, the transformed-transformer method, and the composite method.

The beta-generated (BG) family introduced by [7] has a cumulative distribution function (CDF) given by

(1) $$G\left(x\right)=\int_{0}^{F(x)}b\left(t\right)dt,$$

where b(t) is the probability density function (PDF) of the beta random variable and F(x) is the CDF of any random variable. The PDF corresponding to (1) is given by

(2) $$g\left(x\right)=\frac{1}{B\left(\alpha ,\beta \right)}f\left(x\right)F^{\alpha -1}\left(x\right){\left[1-F(x)\right]}^{\beta -1},\alpha >0,\beta >0;x\in \mathrm{Supp}\left(F\right),$$

where *Supp*(*F*) is the support of *F* and $B\left(\alpha ,\beta \right)=\frac{\Gamma (\alpha )\Gamma (\beta )}{\Gamma \left(\alpha +\beta \right)}.$

Since the proposal of BG family in 2002, several members of the BG family of distributions were investigated. For example, beta-normal [7,8,9], beta-Gumbel [10], beta-Frechet [11], beta-Weibull [8,12,13,14], beta-Pareto [15], beta generalized logistic of type IV [16] and beta-Burr XII [17]. Some extensions of the BG family are also appeared in literature such as Kw-G distribution [18,19], beta type I generalization [20], and generalized gamma-generated family [21].

The beta-generated family of distributions is formed by using the beta distribution in (1) with support between 0 and 1 as a generator. Ref. [22], in turn, were interested whether other distributions with different support can be used as a generator. They extended the family of BG distributions and defined the so called *T*-*X* family. In the *T*-*X* family, the generator b(t) was replaced by a generator $r_{T}\left(t\right),$ where *T* is any random variable with support $\left(a,b\right)$. The CDF of the *T*-*X* family is given by

(3) $$G\left(x\right)=\int_{0}^{W[F(x)]}r\left(t\right)dt,$$

where $W\left[0,1\right]\to$ is a link function that satisfies W(0)→a and W(1)→b. Ref. [23] studied a special case of the *T*-*X* family where the link function, W(.), is a quantile function of a random variable Y. The proposed CDF is defined as

(4) $$F_{X}\left(x\right)=\int_{0}^{Q_{Y}\left[F_{R}\left(x\right)\right]}f_{T}\left(t\right)dt=P\left[T\leq Q_{Y}\left[F_{R}\left(x\right)\right]\right]=F_{T}\left(Q_{Y}\left[F_{R}\left(x\right)\right]\right),$$

where T, R, and *Y* are random variables with CDF $F_{T}\left(x\right)=P\left(T\leq x\right),$ $F_{R}\left(x\right)=P\left(R\leq x\right),$ and $F_{Y}\left(x\right)=P\left(Y\leq x\right).$ The corresponding quantile functions are $Q_{T}\left(p\right),$ $Q_{R}\left(p\right),$ and $Q_{Y}\left(p\right),$ where the quantile function is defined as $Q_{Z}\left(p\right)=\inf\left\{z:F_{Z}\left(z\right)\geq p\right\},0<p<1.$ If densities exist, we denote them by $f_{T}\left(x\right),$ $f_{R}\left(x\right),$ and $f_{Y}\left(x\right).$ Now, if the random variables $T\in \left(a,b\right)$ and $Y\in \left(c,d\right),$ for −∞≤a<b≤∞, and −∞≤c<d≤∞, then the corresponding PDF of (4) is given by

(5) $$f_{X}\left(x\right)=f_{R}\left(x\right)\times \frac{f_{T}\left(Q_{Y}\left[F_{R}\left(x\right)\right]\right)}{f_{Y}\left(Q_{Y}\left[F_{R}\left(x\right)\right]\right)}.$$

If *R* follows the normal distribution $N(\mu ,\sigma^{2})$, then (5) reduces to the *T*-normal family of distributions [24] with PDF given by

(6) $$f_{X}\left(x\right)=\frac{1}{\sigma }\phi \left(\frac{x-\mu }{\sigma }\right)\times \frac{f_{T}\left(Q_{Y}\left[\Phi \left(\frac{x-\mu }{\sigma }\right)\right]\right)}{f_{Y}\left(Q_{Y}\left[\Phi \left(\frac{x-\mu }{\sigma }\right)\right]\right)},$$

where ϕ(.) and Φ(.) are the PDF and CDF of the standard normal distribution, respectively.

The *T*-normal family is a general base for generating many different generalizations of the normal distribution. The distributions generated from the *T*-normal family can be symmetric, skewed to right, skewed to the left, or bimodal. Some of the existing generalizations of normal distribution can be obtained using this framework. In particular, some generalizations of the normal distribution are beta-normal [7], Kumaraswamy normal [19] and gamma-normal distribution [25].

Other generalizations of the normal distribution is the skew-normal, first considered by [26], and it is defined as

$$f_{X}\left(x\right)=2\phi \left(x\right)\Phi \left(\lambda x\right),\;\;x\in R,\;\lambda \in R.$$

Another generalization of the normal distribution is the power-normal distribution [27] with CDF given by

$$F\left(x\right)={\left(\Phi (x)\right)}^{\alpha },\;\;x\in R,\;\alpha \in R^{{}^{+}}.$$

Several properties of the power-normal distribution are studied by [27]. Recently, Ref. [28] proposed a new extension of the normal distribution.

The rest of the paper is organized as follows. In Section 2, we introduce a class of skew-symmetric model by using the logistic kernel and the normal distribution as the baseline distribution. In Section 3, we discuss some structural properties of the logistic-normal (henceforth, LN in short) distribution including moments, tail behavior, and modes. In Section 4, the maximum likelihood estimation method is considered to estimate the model parameters, and a small simulation study is implemented to evaluate the performance of the method. In Section 5, a generalized normal regression model based on skew-LN distribution is developed. In Section 6, applications to two real datasets are given to demonstrate the flexibility and the usefulness of the new distribution and its regression model. We conclude this paper by providing some concluding remarks in Section 7.

## 2. The Symmetric Logistic-G Family of Distributions

If *T* follows the logistic distribution with PDF $f_{T}\left(x\right)=\lambda e^{-\lambda x}{\left(1+e^{-\lambda x}\right)}^{-2},\;\lambda >0$ and *Y* follows the standard logistic distribution (λ=1), then Equation (4) reduces to the Logistic-*G* family of distributions with CDF given by

(7) $$F_{G}\left(x\right)=\frac{G^{\lambda }\left(x\right)}{G^{\lambda }\left(x\right)+{\left(1-G(x)\right)}^{\lambda }},\;\lambda >0;x\in Supp\left(G\right),$$

where G(.) is the CDF of any baseline probability density function. A special case of (7) was studied in some details in [29]. The corresponding PDF of (7) is given by

(8) $$f_{G}\left(x\right)=\lambda g\left(x\right)G^{\lambda -1}\left(x\right)\frac{{\left(1-G\left(x\right)\right)}^{\lambda -1}}{{\left[G^{\lambda }\left(x\right)+{\left(1-G\left(x\right)\right)}^{\lambda }\right]}^{2}},$$

where g(.) is the PDF of G(.).

**Remark** **1.** *The Logistic-G family possesses the following properties*

*i.*  *If g(x) in (8) is a symmetric PDF about μ, then the resulting $f_{G}\left(x\right)$ is a symmetric PDF about μ. i.e., the Logistic-G family in (7) preserves the symmetry property.*
*ii.*  *If a random variable T follows the logistic distribution with scale parameter λ, then the random variable $X=G^{-1}\left(\frac{e^{T}}{1+e^{T}}\right)$ follows the Logistic-G family in (7).*
*iii.*  *The quantile function of the Logistic-G family can be written as*
  (9) $$Q_{G}\left(p\right)=G^{-1}\left[{\left(1+{\left(p^{-1}-1\right)}^{1/\lambda }\right)}^{-1}\right],\;0<p<1.$$

**Figure 1:**
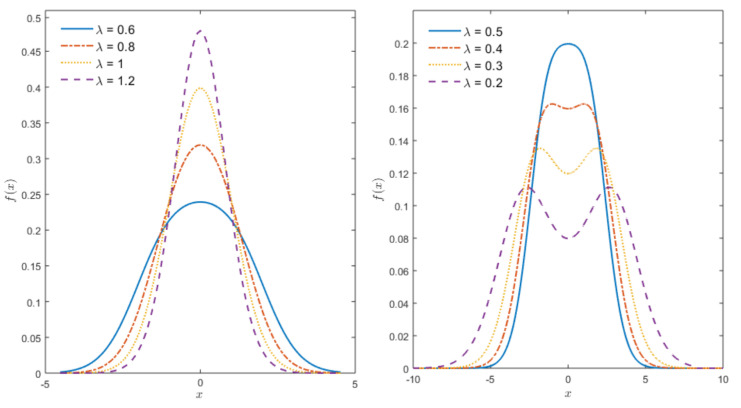
The logistic-normal (LN) density for μ=0,σ=1, and various values of λ.

**Figure 2:**
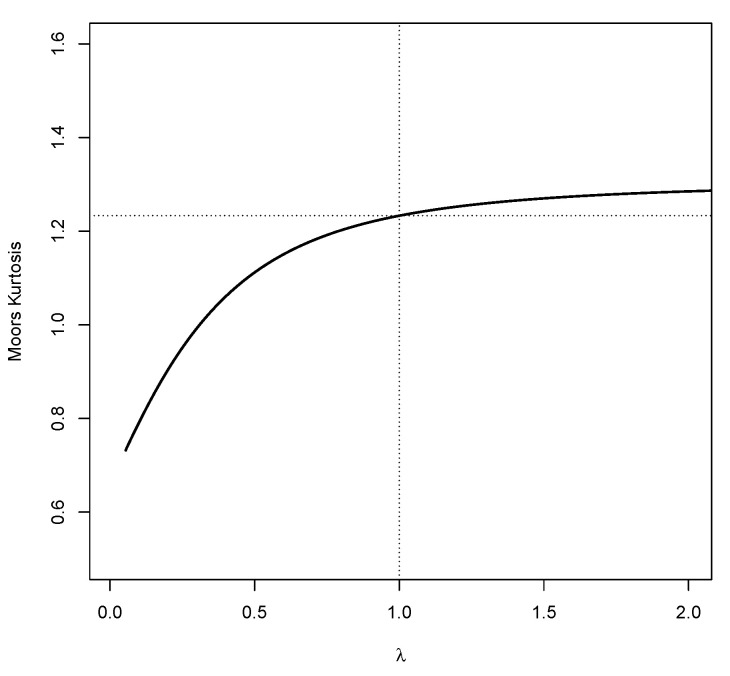
Plot of Moore’s kurtosis of LN distribution for various value of λ. The dashed line represents the Moore’s kurtosis of the standard normal distribution.

Now setting G(x) to be the normal CDF with parameters μ and $\sigma^{2}$, say $G\left(x\right)=\Phi \left(\frac{x-\mu }{\sigma }\right)$, then the Logistic-*G* family reduces to the Logistic-normal distribution with CDF given by

(10) $$F_{N}\left(x\right)=\frac{\Phi^{\lambda }\left(\frac{x-\mu }{\sigma }\right)}{\Phi^{\lambda }\left(\frac{x-\mu }{\sigma }\right)+{\left(1-\Phi \left(\frac{x-\mu }{\sigma }\right)\right)}^{\lambda }},\;\;\;\;\;x\in R,$$

where λ>0,σ>0, and −∞<μ<∞. The associated PDF of (10) is

(11) $$f_{N}\left(x\right)=\frac{\lambda \phi \left(\frac{x-\mu }{\sigma }\right)\Phi^{\lambda -1}\left(\frac{x-\mu }{\sigma }\right){\left(\Phi \left(\frac{x-\mu }{\sigma }\right)\right)}^{\lambda -1}}{\sigma {\left[\Phi^{\lambda }\left(\frac{x-\mu }{\sigma }\right)+{\left(1-\Phi \left(\frac{x-\mu }{\sigma }\right)\right)}^{\lambda }\right]}^{2}},\;x\in R.$$

when λ=1, the logistic-normal (LN(μ,σ,λ), henceforth in short) in (10) reduces to the normal distribution. Thus LN distribution is a generalization of the normal distribution. Furthermore, the LN distribution is a member of the T-normal family proposed by [24]. In Figure 1, graphs of standard LN distribution (where μ=0,σ=1) for various values of λ are provided. Figure 1 shows that the logistic-normal PDF has several advantages, the parameter λ introduces the flexibility on kurtosis (see also Figure 2) and controls whether the distribution is unimodal or bimodal. Moreover, it appears that the bi-modality occurs when λ is approximately less than 0.5.

## 3. Some Properties of LN Distribution

We begin our discussion by providing some useful remarks as listed below.

**Remark** **2.** *Using (10), (11) and Remark 1, the following useful properties can be obtained*

*(i)*  *It is easy to show from (11) that $f_{N}\left(x+\mu \right)=f_{N}\left(\mu -x\right)$ which implies that the LN$\left(\lambda ,\mu ,\sigma \right)$ is symmetric about the location parameter μ.*
*(ii)*  *The mean and median of the LN distribution are μ which is the location parameter of the normal distribution.*
*(iii)*  *The quantile function of the LN distribution can be written as*
  $$Q\left(p\right)=\mu +\sigma \Phi^{-1}\left[{\left(1+{\left(p^{-1}-1\right)}^{1/\lambda }\right)}^{-1}\right],\;0<p<1.$$
*(iv)*  *In order to generate random sample from the LN distribution, first simulate random sample, $t_{i},\;i=1,2,\cdots ,n,$ from logistic(λ) distribution and then compute $x_{i}=\mu +\sigma \Phi^{-1}\left(\frac{e^{t_{i}}}{1+e^{t_{i}}}\right)$.*

**Remark** **3.** Using the fact that $\phi^{\prime }\left(x\right)=-x\phi \left(x\right)$ and setting the derivative of $\log f_{N}\left(x\right)$ in (11) to 0, one can show that Mode(s) of the LN distribution is/are at the point(s) $x_{*}=\mu +\sigma z_{*}$, where $z_{*}$ satisfies the equation

(12) $$z=\frac{\phi (z)}{1-\Phi (z)}\left[\frac{\left(\lambda -1)[1-2\Phi (z)\right]}{\Phi (z)}-\frac{2\lambda \Phi^{\lambda -1}\left(z\right)}{\Phi^{\lambda }\left(z\right)+{\left(1-\Phi (z)\right)}^{\lambda }}+2\lambda \right],\;\;z\in R.$$

From Remark 3, it is easy to see that 0 satisfies Equation (12). Therefore $f_{N}\left(x\right)$ has a critical point at x=μ. We were able to observe numerically that for λ>0.5 the distribution is always unimodal and hence, x=μ is the unique mode in this case. In addition, because of the fact that LN distribution is symmetric about μ for all values of λ, then for the bimodal case, if x=a<μ is a mode then the second mode will be at x=2μ−a.

The tail behaviour of the standard LN distribution (μ=0 and σ=1) as x→±∞ are discussed in the following Lemma.

**Lemma** **1.** *Let Z∈LN(0,1,λ), then as z→±∞,*

$$f_{N}\left(z\right)∼\frac{\exp\left(-\lambda z^{2}/2\right)}{{\left|z\right|}^{\lambda -1}},\;\lambda >0.$$

**Proof.**  As z→∞, $\phi \left(z\right)∼\exp\left(-z^{2}/2\right),$ and $1-\Phi \left(z\right)∼\frac{\phi (z)}{z}$ (see [17]). Consequently, as x→∞, $f_{N}\left(z\right)=\frac{\lambda \phi \left(z\right)\Phi^{\lambda -1}\left(z\right){\left[1-\Phi \left(z\right)\right]}^{\lambda -1}}{{\left(\Phi^{\lambda }\left(z\right)+{\left(1-\Phi (z)\right)}^{\lambda }\right)}^{2}}$ $∼\phi \left(z\right){\left(\frac{\phi (z)}{z}\right)}^{\lambda -1}∼\frac{\exp\left(-\lambda x^{2}/2\right)}{z^{\lambda -1}}$. Similarly, as z→−∞, $f_{N}\left(z\right)∼\frac{e^{-\lambda z^{2}/2}}{{\left|z\right|}^{\lambda -1}}.$ □

Lemma 1 implies that as Z→±∞, the tails of the standard LN distribution behave in similar way as the right tail of the function $\frac{\exp\left(-\lambda x^{2}/2\right)}{x^{\lambda -1}}.$ Note that when 0<λ<1, the tails of $f_{N}\left(x\right)$ approaches 0 slowly, while for λ>1, the tails of $f_{N}\left(x\right)$ approaches 0 faster, meaning that the tail weight increases for higher values of λ. A graphical representation of the association between the tail weight of LN and λ can be shown using the measure of Kurtosis defined by [30]. The Moore’s kurtosis is defined as

(13) $$\gamma_{M}=\frac{Q(7/8)-Q(5/8)+Q(3/8)+Q(1/8)}{Q(6/8)-Q(2/8)}.$$

The values of Moore’s kurtosis of LN(0,1,λ) for various value of λ is depicted in Figure 2. It shows that as λ increases the Moore’s kurtosis increases. For 0<λ<1, there is a sharp change in the kurtosis, while for λ>1 the change is gradual. Figure 1 indicates that for λ<1, the tails of LN distribution are lighter than that of the normal distribution, while for λ>1 the tails of LN distribution are heavier than that of the normal distribution.

### Moments of LN Distribution

Using Remark 2 (ii), the *r*th moment of the LN distribution can be written as $E\left(X^{r}\right)=E{\left(\sigma \Phi^{-1}\left(\frac{e^{T}}{1+e^{T}}\right)+\mu \right)}^{r},$ where the random variable *T* follows the logistic distribution with scale parameter λ. Therefore,

$$E\left(X^{r}\right)=\lambda \int_{-\infty }^{\infty }{\left(\sigma \Phi^{-1}\left(\frac{e^{t}}{1+e^{t}}\right)+\mu \right)}^{r}e^{\lambda t}{\left(1+e^{\lambda t}\right)}^{-2}dt.$$

Now, $\Phi^{-1}\left(x\right)=\sqrt{2}\;{\mathrm{erf}}^{-1}\left(2x-1\right),$ where $\mathrm{erf}\left(x\right)=\frac{2}{\sqrt{\pi }}\int_{0}^{x}e^{(}-t^{2})dt.$ This implies that

EXr=Eσ2erf−11−21+eT−1+μr=∑j=0rrj2j/2σjμr−jξj,

where $\xi_{j}=\lambda \int_{-\infty }^{\infty }\left[{\mathrm{erf}}^{-1}\left(1-2{\left(1+e^{t}\right)}^{-1}\right)\right]e^{\lambda t}{\left(1+e^{\lambda t}\right)}^{-2}dt.$

$\xi_{j}$ can be evaluated using numerical integration from any available software such as *R* or SAS.

**Remark** **4.** *Let $X∼LN\left(\mu ,\sigma ,\lambda \right)$, then*

*i.*  *From Remark 2 (i), the rth central moment $E{\left(X-\mu \right)}^{r}=0$ for any odd integer r.*
*ii.*  *$X∼LN\left(\mu ,\sigma ,\lambda \right)$ implies that X=σZ+μ where Z∼LN(0,1,λ). Therefore,*
  E(Xr)=∑k=0rrkσkμr−kE(Zk)=∑evenkrrjσkμr−kE(Zk).

## 4. Estimation and Simulation

In this section, the maximum likelihood method (MLE) is used to estimate the parameters of LN distribution. Moreover, a small simulation study is performed to assess the performance of the MLE method.

### 4.1. Parameter Estimation of LN Distribution

Let $x_{1},x_{2},\cdots ,x_{n}$ be a random sample of size *n* taken from LN distribution. Then the log-likelihood function is given by

(14) $$\begin{array}{cc} \ell (\lambda ,\mu ,\sigma )= & n\log\left(\frac{\lambda }{\sigma }\right)+\sum_{i=1}^{n}\log\phi \left(\frac{x_{i}-\mu }{\sigma }\right)+\left(\lambda -1\right)\sum_{i=1}^{n}\log\Phi \left(\frac{x_{i}-\mu }{\sigma }\right)\\ & +\left(\lambda -1\right)\sum_{i=1}^{n}\log\left(1-\Phi \left(\frac{x_{i}-\mu }{\sigma }\right)\right)\\ & -2\sum_{i=1}^{n}\log\left[\Phi^{\lambda }\left(\frac{x-\mu }{\sigma }\right)+{\left(1-\Phi \left(\frac{x-\mu }{\sigma }\right)\right)}^{\lambda }\right]. \end{array}$$

The MLE of $\hat{\lambda },$ $\hat{\mu },$ and $\hat{\sigma }$ of the parameters λ,μ, and σ can be obtained by maximizing numerically the log-likelihood function in (14). The initial value of μ is taken to be the moment estimator $\bar{x}.$ The initial value of σ is taken to be the sample standard deviation, s. To obtain the initial value of the parameter λ, we use Remark 2 (iv) as follows; assume the random sample $t_{i}=\log\left[\frac{\Phi \left(\frac{x_{i}-\bar{x}}{s}\right)}{1-\Phi \left(\frac{x_{i}-\bar{x}}{s}\right)}\right],\;i=1,2,...,n$ is taken from the logistic distribution with parameter λ. By equating the population variance $\frac{\pi^{2}}{3\lambda^{2}}$ of logistic distribution with the sample variance, $s_{T}^{2}$ of the random sample $t_{i}$ and solving it for λ, we obtain $\lambda_{0}=\frac{\sqrt{\frac{1}{3}\pi }}{s_{T}}.$

The trust-region optimization routine in SAS (PROC IML and CALL NLPTR) is used in order to maximize the likelihood function in (14). The trust-region optimization routine is a powerful technique that can optimize complicated functions. It outputs the iteration details including parameter estimates, their standard errors, and the value of the gradient function at which iteration stops.

### 4.2. Simulation

In order to evaluate the performance of the ML method, a small simulation study is conducted with sample sizes n=30,50,70 and with three different parameter combinations. The study involved computing and analyzing the relative bias [(Estimate-Actual)/Actual] and the standard deviation of the estimates. The results of the study are reported in Table 1.

From Table 1, it is observed that the ML estimate of the parameter μ is overestimated. Moreover, when λ<1, the ML estimates of λ and σ are overestimated. On the other hand, when λ>1, ML estimates of λ and σ are underestimated. Moreover, for small sample size(s) and when λ<1, MLE method does not perform well. In fact, standard deviations are higher than the corresponding estimated values. However, the results for higher sample sizes and when λ>1, it can be seen that the MLE method performs quite well in estimating the model parameters.

## 5. Skew-LN and Its Generalized Normal Regression Model

In this section, we first propose a skewed type of LN distribution that can be used to fit skewed dataset. In Section 5.2, we propose a location-scale regression model based on the skew-LN distribution.

### 5.1. Skew Logistic-Normal Distribution

For skewed data, one can generate a skew-LN distribution in various ways. Once way is by exponentiating the CDF of the LN distribution as

(15) $$F\left(x\right)={\left[\frac{\Phi^{\lambda }\left(\frac{x-\mu }{\sigma }\right)}{\Phi^{\lambda }\left(\frac{x-\mu }{\sigma }\right)+{\left(1-\Phi \left(\frac{x-\mu }{\sigma }\right)\right)}^{\lambda }}\right]}^{\alpha },\;\;\alpha >0,\lambda >0,\;\;\;x\in R.$$

Note that when α=1, the skew-LN distribution in (15) reduces to LN distribution. Moreover, when λ=1, the skew-LN reduces to the eponentiated-normal distribution proposed by [27]. Finally, when α=λ=1, the skew-LN distribution reduces to normal distribution.

**Figure 3:**
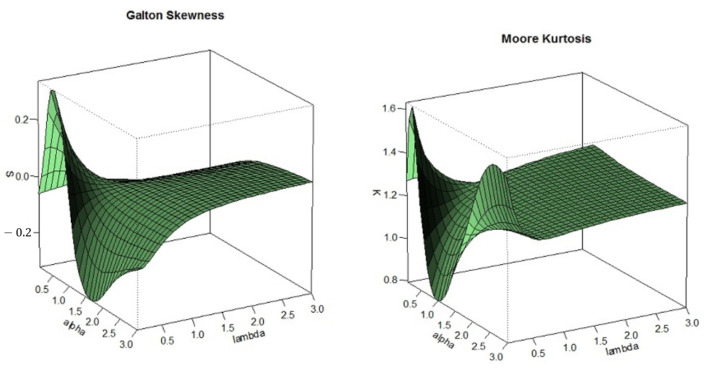
Three-dimensional plots of Galton’s skewness and Moore’s kurtosis for various values of α and λ.

In order to analyze the skewness and kurtosis regions of the skew-LN distribution, the Refs. [30,31] measures were plotted against the parameter α and λ. Figure 3 shows that the distribution is right skewed for α,λ<1 and left skewed for α>1,λ<1 and α<1, λ>1. The plot of kurtosis in Figure 3 demonstrates the flexibility of the proposed distribution. For λ<1, the tails of the skewed LN can be heavier or lighter than that tail of the normal distribution.

The skew-LN distribution has several advantages; the parameter α introduces the flexibility on the skewness and the parameter λ introduces the flexibility on the kurtosis. Furthermore, the main advantage of the skew-LN when compared with Azzalini skew-normal is the flexibility of fitting data with wider range of skewness and kurtosis. Based on numerical calculations, for the Azzalini skew-normal, the Galton’s skewness ranges between −0.1443 and 0.1443 and the Moor’s kurtosis ranges between 1.1746 and 1.2460. However, for the skew-LN, the Galton’s skewness ranges between −0.3000 and 0.3000 and the Moor’s kurtosis ranges between 0.8000 and 1.6000. It is also worth mentioning that the skew-LN can be unimodal or bimodal and has closed form CDF which is not the case of Azzalini skew-normal distribution.

### 5.2. Generalized Normal Regression Model Based on Skew-LN Distribution

The traditional linear regression model that assumes normal residuals is applied extensively in engineering and science. However, the normality assumption of the model residuals is often ineffective. This drawback can be overcome by using a generalized normal regression model that assumes non-normal response Y. In this section, *T* is assumed to follow the skew-LN distribution. The following location-scale regression model is considered based on the skew-LN distribution

(16) $$y_{i}={\underline{x}}_{i}^{T}\underline{\beta }+\sigma Z_{i},\;i=1,2,\cdots ,n,$$

where $y_{i}$ pertains to the response variable with a skew-LN distribution in (15), $\underline{\beta }={\left(\beta_{0},\beta_{1},\cdots ,\beta_{p}\right)}^{T},$ and σ>0 are unknown parameters. Every $y_{i}$ has a covariate vector ${\underline{x}}_{i}^{T}=\left(1,x_{i1},\cdots ,x_{ip}\right)$ that models the linear predictor $\mu_{i}={\underline{x}}_{i}^{T}\underline{\beta }.$ The random error $Z_{i}$ follows the skew-LN $\left(0,1,\lambda ,\alpha \right)$ distribution.

**Remark** **5.** *The skew-LN regression model in (16) has several nested regression models. These special cases are enumerated as follows:*

*1.*  *The regression model in (16) is reduced to the traditional normal linear regression model when α=λ=1.*
*2.*  *The exponentiated-normal (Exp-N) regression model is obtained when λ=1. This location-scale regression model is based on the power normal distribution introduced by [27].*
*3.*  *The LN regression model based on the distribution (10) is obtained when α=1.*

A sample of $\left(y_{1},{\underline{x}}_{1}\right),\cdots ,\left(y_{n},{\underline{x}}_{n}\right)$ of *n* independent observations is considered, and the log-likelihood function for model (16) parameters $\underline{\theta }={\left(\lambda ,\alpha ,\sigma ,{\underline{\beta }}^{T}\right)}^{T}$ is presented as

(17) $$\begin{array}{ccc} \ell (\underline{\theta }) & = & n\log\left(\frac{\alpha \lambda }{\sigma }\right)+\sum_{i=1}^{n}\log\phi \left(z_{i}\right)+\left(\lambda \alpha -1\right)\sum_{i=1}^{n}\log\Phi \left(z_{i}\right)\\ & & +\left(\lambda -1\right)\sum_{i=1}^{n}\log\left[1-\Phi (z_{i})\right]\\ & & -\left(\alpha +1\right)\sum_{i=1}^{n}\log\left[\Phi^{\lambda }\left(z_{i}\right)+{\left(1-\Phi (z_{i})\right)}^{\lambda }\right], \end{array}$$

where $z_{i}=\frac{y_{i}-{\underline{x}}_{i}^{T}\underline{\beta }}{\sigma }.$ The maximum likelihood $\hat{\underline{\theta }}$ of the parameter vector $\underline{\theta }$ can be obtained by maximizing the log-likelihood function in (17) numerically.

## 6. Applications

In this section, we apply the LN distribution and the generalized normal regression to two real-life datasets. The first dataset possesses a bimodal shape, and the fit of the LN distribution is compared with the mixture normal distribution. For the second application, the skew-normal regression model is compared with some nested sub-models and some other generalization of the normal regression models. Maximum likelihood method is used to estimate the model parameters.

### 6.1. Fitting LN Distribution to Buoys Data

**Figure 4:**
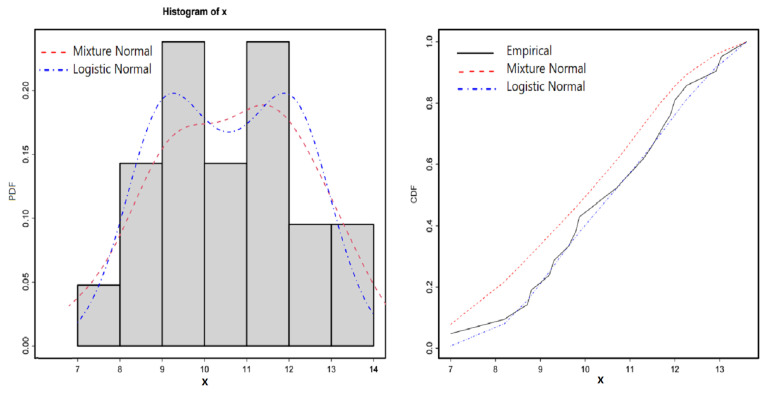
Plots of fitted distributions for the Buoys dataset.

In this subsection the LN distribution is fitted to a bimodal datasets using ML method. The dataset is obtained from National Data Buoy Center (NDBC). It represents the number of buoys situated in the North East Pacific: Buoy 46,005 (46 *N*, 131 *W*) for the time period 1 January 1983 to 31 December 2003. The data is available from [1]. The Histogram in Figure 4 shows that the distribution of the data possesses a bimodality shape, for this reason, we fitted the dataset to both LN and the mixture normal distributions. The results of the maximum likelihood estimates, the log-likelihood value, the AIC (Akaike Information Criterion) and the Kolmogorov-Smirnov (K-S) test statistic for the fitted distributions are reported in Table 2. Figure 4 displays both the empirical and the fitted cumulative distribution as well as the probability density functions for the fitted distributions. The results in Table 2 indicate that the LN distribution outperforms the mixture normal distribution. In fact, the fitted CDF in Figure 4 shows that the mixture normal distribution does not provide an adequate fit. The fact that the LN distribution has only three parameters adds an extra advantage to the distribution over the mixture normal distribution.

### 6.2. Modeling Real Estate Valuation Using the Generalized Normal Regression Model

The dataset contains historical data on the real estate market from June 2012 to May 2013. The data is obtained from Sindian District in New Taipei City, Taiwan (for additional details, see [32]). The data consist of n=414 transaction records of real estate property. The data can be used to establish the relationship between housing price (per unit area) and its predictive regressors. The following variables are used (for i=1,2,⋯,414). Response variable y is the housing price per unit area (10,000 New Taiwan Dollar/Ping, where 1 Ping = 3.3 m${}^{2}$), the covariates are as follows: $x_{i1}$ is the transaction date (e.g., 2013.250=2013 March and 2013.500=2013 June), $x_{i2}$ is the house age (in years), $x_{i3}$ is the distance to the nearest MRT station (in meters), $x_{i4}$ is the number of convenience stores in the living circle on foot (integer), and $x_{i5}$ is the geographic coordinate, latitude (in degrees). The data are analyzed on the basis of the following skew-LN regression model

$$y_{i}=\beta_{0}+\beta_{1}x_{i1}^{*}+\beta_{2}x_{i2}^{*}+\beta_{3}x_{i3}^{*}+\beta_{4}x_{i4}^{*}+\beta_{5}x_{i5}^{*}+\sigma Z_{i},\;\;\;\;\;\;i=1,\ldots ,414,$$

where the error terms $Z_{i}$ are independent random variables that assumed to follow the skew-LN$\left(0,1,\lambda ,\alpha \right)$ distribution, and $x_{ij}^{*}=\left(x_{ij}-{\bar{x}}_{j}\right)/s_{j},j=1,2,\ldots ,5$, are the standardized covariates, which are considered because of the fact that some covariates are measured using different scales. Additionally, the fit under the skew-LN regression model is compared with several regression models, including the regression model based on the beta-normal (BN) distribution [7], the regression model based on the skewed-normal (SN) distribution [26], and the extended normal (EN) regression model [28]. Furthermore, the skew-LN regression model is compared with its nested models, including LN, Exp-N, and normal regression. In this application, the model parameters are estimated using the maximum likelihood method and SAS programming language is used. The initial values of $\beta_{0},\cdots ,\beta_{5}$ and σ are obtained from fitting the data to the normal regression model. The initial values of the other parameters are set to 1. Table 3 shows the MLEs results of fitting skew-LN, LN, Exp-N, SN, EN, and normal regression models to the data.

The fitted skew-LN an LN regression models show that the estimates $\beta_{0},\cdots ,\beta_{5}$ and σ are significant at 5% level of error. Table 4 presents the goodness of fit statistics including AIC, consistent AIC (AICC) and Bayesian information criterion (BIC). The goodness of fit statistics show that the skew-LN regression model outperforms the other regression models. We also notice that the LN regression model has the second-lowest values of AIC, AICC, and BIC. Hence, skew-LN and LN regression models can be used effectively to analyze the real estate valuation data.

The likelihood ratio (LR) statistic is utilized to compare the skew-LN regression model with its sub-models; normal, LN, and Exp-N regression models. The LR test statistic values and the corresponding p-values are given in Table 5. This Table shows that the skew-LN regression model has a better fit when compared with the other sub-models. The LN regression model also has a better fit when compared with the normal regression model.

## 7. Concluding Remarks

In this paper, two generalizations of the normal distribution namely; logistic-normal and skew logistic-normal distributions were investigated. Several mathematical and structural properties have been studied such as shape properties. The proposed generalizations of the normal distribution exhibit a great flexibility in modeling symmetric as well as skewed datasets. Moreover, new regression models based on both logistic-normal and skew logistic-normal were developed. Two real datasets were used to illustrate the applicability of the distributions and their regression models.

Future work could be devoted toward investigating other parameter estimation methods for the LN and the skew-LN distributions. The applicability of the skew-LN regression model to other fields could be further explored.

## Figures and Tables

**Table 1:** Relative bias and standard deviation of the maximum likelihood method (MLE) for LN distribution.

Sample Size	Actual Value			Relative Bias			Standard Deviation		
n	λ	μ	σ	$\hat{\lambda }$	$\hat{\mu }$	$\hat{\sigma }$	$\hat{\lambda }$	$\hat{\mu }$	$\hat{\sigma }$
30	0.5	2	1	1.2698	0.0276	0.8064	1.5928	0.2797	1.8965
50				0.6606	0.0256	0.4205	0.4651	0.2749	0.5992
70				0.3290	0.0140	0.1422	0.4005	0.2013	0.4485
30	1.5	2	1	−0.1422	0.0101	−0.1210	0.7309	0.1224	0.4959
50				−0.0692	0.0339	−0.1074	0.5927	0.1005	0.3494
70				−0.0671	0.0087	−0.0898	0.3460	0.0773	0.2153
30	2	3	1	−0.3089	0.0113	−0.3005	0.8190	0.0978	0.3418
50				−0.3247	0.0083	−0.2915	0.8095	0.0827	0.2212
70				−0.3162	0.0076	−0.2990	0.8007	0.0575	0.1379

**Table 2:** Estimates of the parameters and goodness of fit measures for the Buoys data.

Distribution	LN	Mixture Normal
		$\hat{\lambda }=0.5515\left(0.2920\right)$
	$\hat{\lambda }=0.2734\left(0.3304\right)$	$\hat{\mu_{1}}=8.6051\left(0.6836\right)$
Parameter Estimates	$\hat{\mu }=10.57\left(0.3145\right)$	$\hat{\mu_{2}}=11.4634\left(0.6930\right)$
	$\hat{\sigma }=0.6507\left(0.5051\right)$	$\hat{\sigma_{1}}=1.4994\left(0.7293\right)$
		$\hat{\sigma_{2}}=1.0750\left(0.3770\right)$
Log-likelihood	80.7	109.5
AIC	86.7	119.5
K-S	0.2273	0.6901

**Table 3:** MLEs of the parameters (SEs in parentheses) and p-values below SE for the real estate valuation data.

Model	Estimates										
	λ	α	a	b	σ	$\beta_{0}$	$\beta_{1}$	$\beta_{2}$	$\beta_{3}$	$\beta_{4}$	$\beta_{5}$
Skew-LN	201.56 (11.3874) <0.0001	2.4560 (0.5421) <0.0001	-	-	1710.11 (1.3422) <0.0001	31.1581 (1.6737) <0.0001	0.7992 (0.3525) 0.0239	−3.3125 (0.3765) <0.0001	−5.0717 (0.5341) <0.0001	3.7339 (0.4609) <0.0001	2.6738 (0.4435) <0.0001
LN	251.26 (10.1697) <0.0001	-	-	-	1742.09 (1.4668) <0.0001	37.3380 (0.3675) <0.0001	1.0021 (0.3645) 0.0062	−3.3672 (0.3825) <0.0001	−5.0580 (0.5408) <0.0001	3.6095 (0.4784) <0.0001	2.9054 (0.4900) <0.0001
Exp-N	-	-	-	32.1513 (20.3311) 0.1146	16.9758 (1.7343) <0.0001	2.9407 (7.8762) 0.7091	0.8789 (0.3932) 0.0259	−3.1964 (0.4112) <0.0001	−5.0716 (0.5809) <0.0001	3.4727 (0.4988) <0.0001	2.8686 (0.4746) <0.0001
EN	-	-	1.3218 (1.7053) 0.4387	35.3119 (27.6631) 0.2025	19.2211 (11.1653) 0.0859	5.8752 (16.5371) 0.7226	0.8829 (0.3936) 0.0254	−3.1970 (0.4110) <0.0001	−5.0767 (0.5812) <0.0001	3.4745 (0.4987) <0.0001	2.8644 (0.4751) <0.0001
BN	-	-	111.13 (237.23) 0.6397	1.5116 (0.7873) 0.0556	23.9265 (11.4859) 0.0379	−17.7314 (39.9566) 0.6574	0.8863 (0.3943) 0.0251	−3.1978 (0.4112) <0.0001	−5.0561 (0.5789) <0.0001	3.4673 (0.4993) <0.0001	2.8947 (0.4774) <0.0001
SN	2.3462 (0.2908) <0.0001	-	-	-	12.4444 (0.5943) <0.0001	29.1849 (0.5737) <0.0001	0.9097 (0.3949) 0.0217	−3.1574 (0.4120) <0.0001	−5.1793 (0.5934) <0.0001	3.5278 (0.5015) <0.0001	2.7417 (0.4729) <0.0001
N	-	-	-	-	8.7832 (0.3052) <0.0001	37.9803 (0.4317) <0.0001	1.4478 (0.4352) 0.0010	−3.0689 (0.4350) <0.0001	−5.4944 (0.6138) <0.0001	3.3466 (0.5486) <0.0001	2.8156 (0.5442) <0.0001

**Table 4:** Goodness of fit statistics for the real estate valuation data.

Model	−ℓ	AIC	AICC	BIC
Skew-LN	1433.5327	2885.0654	2885.5109	2921.2982
LN	1444.2295	2904.4590	2904.8146	2936.6659
Exp-N	1454.7811	2925.5622	2925.9178	2957.7691
EN	1454.7551	2927.5102	2927.9557	2963.7430
BN	1454.3973	2926.7946	2927.2401	2963.0274
SN	1458.6946	2933.3892	2933.7448	2965.5961
N	1486.9953	2987.9906	2988.2665	3016.1717

**Table 5:** LR statistics for the real estate valuation data.

	Hypotheses	LR Statistic	*p*-Value
Skew-LN vs. LN	$H_{0}:\alpha =1$	21.3936	<0.0001
Skew-LN vs. Exp-N	$H_{0}:\lambda =1$	42.4968	<0.0001
Skew-LN vs. Normal	$H_{0}:\alpha =\lambda =1$	106.9252	<0.0001
LN vs. Normal	$H_{0}:\lambda =1$	85.5316	<0.0001
